# Comparative efficacy and safety of high-dose versus low-dose tranexamic acid in adolescent idiopathic scoliosis: A systematic review and meta-analysis

**DOI:** 10.1371/journal.pone.0320391

**Published:** 2025-04-01

**Authors:** Xin Liu, Zhong Ma, Jiangdong An, Zhiqiang Luo

**Affiliations:** 1 Department of Orthopaedics, Lanzhou University Second Hospital, Lanzhou, Gansu, China; 2 Key Laboratory of Bone and Joint Disease Research of Gansu Provincial, Lanzhou, Gansu, China; Stony Brook University School of Medicine, UNITED STATES OF AMERICA

## Abstract

**Objective:**

The objective of this meta-analysis was to evaluate the comparative effectiveness and safety of high-dose versus low-dose tranexamic acid (TXA) in adolescents undergoing treatment for idiopathic scoliosis.

**Methods:**

A comprehensive literature search was conducted across PubMed, Web of Science, Embase, Cochrane Library, and China National Knowledge Infrastructure (CNKI) databases up to March 2024. We sought to identify randomized controlled trials (RCTs) and retrospective controlled studies (RCSs) assessing the impact of high-dose compared to low-dose TXA on perioperative blood loss and transfusion requirements in spinal fusion procedures for adolescent idiopathic scoliosis. The study was registered in INPLASY (Registration number: INPLASY202480018).

**Results:**

Our meta-analysis included data from six studies: two high-quality RCTs and four lower-quality RCSs, comprising a total of 611 participants. Subgroup analysis revealed that high-dose TXA significantly reduced intraoperative blood loss and transfusion rates in RCSs, whereas no significant differences were observed in RCTs. The combined findings showed that high-dose TXA was associated with a significant reduction in intraoperative blood loss [weighted mean difference (WMD) =  -215.48, 95% confidence interval (CI) (-367.58, -63.37), P <  0.001], as well as a decreased likelihood of transfusion [risk ratio (RR) =  0.40, 95% CI (0.30, 0.53), P <  0.001]. Operative time did not differ significantly, and no thromboembolic events were reported in either treatment group. The differences between high and low doses varied widely across studies.

**Conclusion:**

This meta-analysis indicates that high-dose TXA does not significantly reduce intraoperative blood loss, transfusion rates, or operative time compared to low-dose TXA in adolescent idiopathic scoliosis. While RCSs showed some benefit, our analysis places more emphasis on the results from RCTs, which did not show significant differences. Further high-quality RCTs are needed to confirm its effectiveness and safety.

## Introduction

Adolescent Idiopathic Scoliosis (AIS) is characterized by an enigmatic lateral and rotational spinal deformity that emerges during puberty, typically involving several vertebrae and potentially accompanied by thoracic abnormalities [1,2]. Spinal fusion surgery represents a cornerstone surgical intervention for AIS, entailing a dorsal incision to expose the spinal column and the subsequent utilization of internal fixation devices, such as rods and screws, to rectify and stabilize the deformed spine, thereby facilitating vertebral fusion [3]. This intricate procedure necessitates meticulous vertebral osteotomies and soft tissue dissection, rendering it highly complex and associated with substantial trauma. The extended duration of the surgery and the extensive nature of the surgical incisions often result in considerable intraoperative hemorrhage, potentially leading to postoperative anemia, prolonged recovery periods, and an increased risk of subsequent complications [4,5]. Effective perioperative blood management is thus imperative in AIS spinal fusion surgeries [6]. The primary objectives of blood management include the maximal reduction of intraoperative blood loss (IBL) and the need for transfusions to mitigate the risks associated with transfusion-related complications, such as infectious diseases, acute lung injury, and hemolytic reactions [7,8]. Identifying efficacious hemostatic strategies is therefore a critical aspect of clinical management in this context.

Tranexamic Acid (TXA) is an antifibrinolytic medication that has been substantiated to effectively curtail hemorrhage in surgical contexts, by hindering plasmin activity and thus impeding fibrinolysis, which facilitates the stabilization of blood clots [9]. The utilization of TXA in various surgical modalities, including spinal procedures, has been associated with a substantial reduction in IBL and diminished transfusion requirements [10–12]. Nevertheless, the debate persists concerning the optimal dosing of TXA within the scope of spinal fusion surgeries for AIS [13]. Evidence from certain studies indicates that higher doses of TXA may surpass lower doses in mitigating hemorrhage and transfusion needs, but concurrently may elevate the incidence of severe side effects, such as thrombotic episodes and epileptic seizures [14]. In light of the limited consensus on high-dose TXA utilization, we have undertaken a systematic meta-analysis aimed at assessing the therapeutic efficacy and safety profile of high versus low-dose TXA in the context of AIS spinal fusion interventions. The outcomes of this inquiry are intended to bolster the evidence foundation for clinical practitioners implementing TXA, enhancing perioperative hematologic management strategies.

## Methods

Literature review and meta-analysis were conducted by the Preferred Reporting Items for Systematic Reviews and Meta-Analyses (PRISMA) statement (S1 Table) [15]. The systematic review protocol is registered in the INPLASY International Registry of Systematic Reviews (Registration number: INPLASY202480018).

### Search strategy

The two authors (XL and ZM) independently searched five databases: PubMed, Web of Science, Embase, Cochrane, and CNKI, covering the period from their inception until March 15, 2024. The main keywords used for the search focus on “tranexamic acid” OR “TXA” in the context of “adolescent idiopathic scoliosis” OR “AIS”. The reference lists of all retrieved articles were thoroughly reviewed to identify additional relevant studies. We have uploaded a complete search strategy example (using PubMed as an example, see S2 Table).

### Study selection

The inclusion criteria for our meta-analysis were as follows: All patients included in the studies had to have undergone corrective surgery for AIS. The studies needed to compare a control group receiving a low-dose TXA with an experimental group receiving a high-dose TXA. The primary endpoints for the analysis were IBL, transfusion rate, operation time, and thromboembolic events. Additionally, the eligible studies were required to be designed as either randomized controlled trials (RCTs) or retrospective controlled studies (RCS). Importantly, the study population had to consist of individuals without any bleeding disorders or a history of antifibrinolytic treatment.

In terms of exclusion criteria, studies focusing on other types of spinal disorders, such as non-idiopathic scoliosis, lumbar disc herniation, and spinal fractures, were excluded from the analysis. Furthermore, articles classified as case reports, reviews, commentaries, or any other types of publications that did not provide extractable data were also excluded. Finally, any duplicate publications were removed to ensure the integrity and originality of the data used in the meta-analysis.

### Data extraction

To ensure the accuracy of data extraction, two researchers (XL and ZM) independently reviewed and extracted the following information: authorship, publication year, country of origin, study design, sample size, participant age, preoperative hemoglobin levels, surgical techniques employed, dosage of TXA administered, criteria for blood transfusion, and outcome measures. If discrepancies arose during data extraction, the two researchers resolved them through discussion and consensus. When a consensus could not be reached, a third researcher (JDA) evaluated the full text, and discrepancies were resolved through group discussion. If necessary, the authors of the included studies were contacted for additional information. The primary outcomes assessed were the IBL, transfusion rate, operation time, and thromboembolic events.

### Quality assessment

Use The Cochrane Risk of Bias Tool 2 (RoB 2) to evaluate RCT studies [16]. The RoB 2 evaluation includes the following steps: randomization process, deviations from intended intervention, missing outcome data, measurement of outcomes, and selection of the reported result. Each domain will be assessed as “low risk of bias,” “some concerns,” or “high risk of bias.” Additionally, the Risk Of Bias In Non-randomized Studies of Interventions (ROBINS-I) tool is used to assess included RCSs [17]. The ROBINS-I tool evaluates studies as having low, moderate, serious, and critical risk of bias. It consists of seven domains: confounding bias, selection bias, classification bias, performance bias, missing data bias, measurement bias, and reporting bias. The quality of evidence will be assessed using the Grading of Recommendations, Assessment, Development, and Evaluations (GRADE) approach [18], and rated as high, moderate, low, or very low. All evaluations will be conducted independently by two researchers (XL and ZM).

### Statistical analysis

For continuous variables (intraoperative blood loss and operation time), we calculated the weighted mean difference (WMD) and 95% confidence intervals (CI). For binary variables (transfusion rates), we calculated the risk ratio (RR) and its 95% CI. Additionally, we reported the risk difference (RD) to provide information on the absolute effect. Heterogeneity was assessed using the Chi-squared test and I^2^ statistic. A fixed-effect model was employed in the absence of significant heterogeneity (I^2^ <  50%, P >  0.1); otherwise, a random-effects model was chosen. Meta-analyses were conducted using RevMan 5.4 for Windows (Cochrane Collaboration, Oxford, UK) and STATA software version 17.0 (Stata Corporation, College Station, Texas, USA).

## Results

### Search result

**Fig 1:**
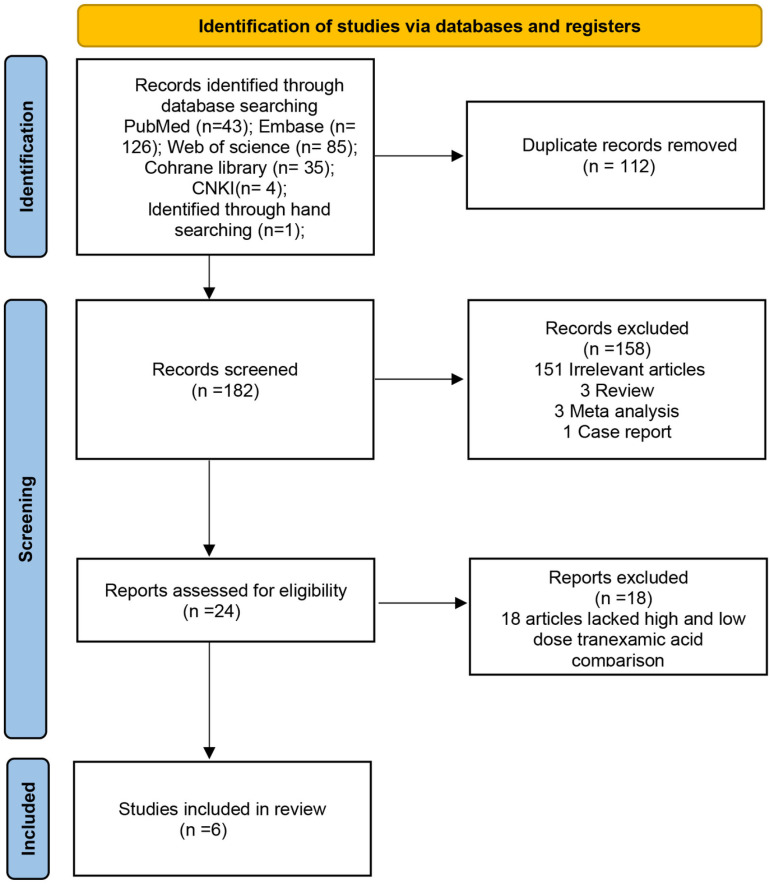
The flow chart of studies selecting.

Our search yielded a total of 294 articles, including 85 from Web of Science, 35 from Cochrane Library, 126 from Embase, 43 from PubMed, 4 from CNKI, and 1 identified through manual search. After duplicates were removed using EndNote (version X9) and an additional 15 duplicate articles were manually excluded, 182 articles remained (a detailed list is provided in S3 Table). A preliminary screening based on titles and abstracts excluded 158 irrelevant articles, leaving 24 studies. Following a full-text review, 18 articles were excluded due to the lack of comparison between high and low doses of TXA, resulting in a total of 6 studies being included in the final meta-analysis [14,19–23] (Fig 1). The data extracted from the included studies are summarized in S4 Table.

### Study characteristics and quality assessment

This meta-analysis incorporated 4 RCSs and 2 RCTs, published between 2009 and 2022. The sample sizes ranged from 11 to 126 participants, culminating in a total of 611 subjects, with 304 in the high-dose TXA group and 307 in the low-dose group (Table 1).

**Table 1:** Characteristics of the included studies.

Author (year)	Country	Study design	No. H VS L	Mean age (years): H VS L	Preoperative Hb (g/dl, H VS L)	Surgical Procedure	Dose of TXA (loading + maintenance) H VS L	Transfusion criteria	Outcome measures
Tumber (2022)	USA	RCS	126/97	14.4/14.0	13.5/13.4	PSF	30 mg/kg + 10 mg/kg/h; <30 mg/kg + 10 mg/kg/h	NR	①②③④
Hasan (2021)	Malaysia	RCT	83/83	14.1/14.6	13.8/13.8	PSF	30 mg/kg + 10 mg/kg/h; 10 mg/kg + 1 mg/kg/h	Hb < 8 g/dL	①②③④
Zhang (2020)	China	RCS	15/15	15.8/14.9	NR	Spinal fusion	100 mg/kg + 10 mg/kg/h; 10 mg/kg + 10 mg/kg/h	NR	①③④
Saleh (2018)	Egypt	RCT	25/25	14.6/14.6	NR	PSF	50 mg/kg + 20 mg/kg/h; 10 mg/kg + 10 mg/kg/h	NR	①③④
Johnson (2017)	USA	RCS	44/72	14.5/14.3	13.5/13.7	PSF	50 mg/kg + 5 mg/kg/h; 10 mg/kg + 1 mg/kg/h	Hb < 9 g/dL	①②③④
Grant (2009)	Canada	RCS	11/15	15.4/ 14.7	13.8/13.4	PSF	20 mg/kg + 10 mg/kg/h; 10 mg/kg + 1 mg/kg/h	Hb < 8 g/dL	②③④

①Intraoperative blood loss; ②Operation time; ③Transfusion rate; ④Thromboembolic events;

PSF: posterior spinal fusion; RCT: randomized controlled trial; RCS: retrospective controlled study; H: high-dose tranexamic acid group; L: low-dose tranexamic acid group; Hb: hemoglobin; NR: no record.

**Fig 2:**
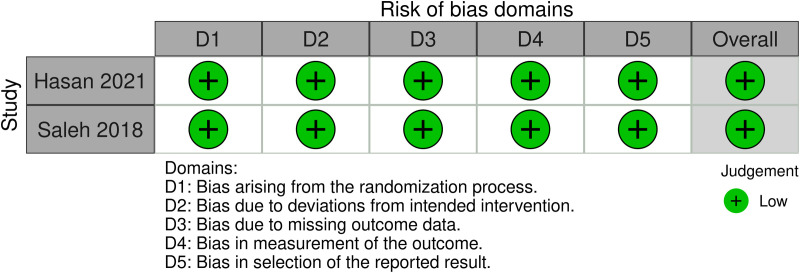
Bias risk assessments for each RCT study.

**Fig 3:**
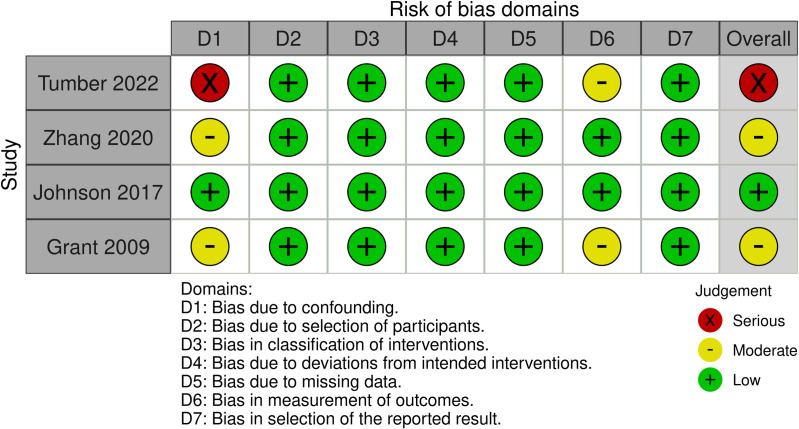
Bias risk assessments for each RCS study.

Fig 2 shows the quality assessment of the RCTs, with both studies considered to have a low risk of bias. Additionally, using the ROBINS-I tool, four RCS studies were assessed: two studies had a moderate risk of bias, one study had a serious risk of bias, and one study had a low risk of bias (Fig 3).

## Results of the meta-analysis

We systematically evaluated IBL, transfusion rate, and operation time. Table 2 summarizes the effect size, confidence intervals, and certainty of the evidence for each outcome. Given the quality of evidence, we downgraded the GRADE rating due to several key factors. First, several of the RCSs were assessed as high risk of bias, mainly due to potential selection and information bias. Second, there was significant heterogeneity across the studies (I^2^ >  50%), which increased uncertainty in the findings. Third, some of the studies included small sample sizes, which may have impacted the stability and generalizability of the results. These three factors contributed to the downgrading of the evidence quality in our analysis.

**Table 2:** Summary of results.

Outcome	Effect Size	95% Confidence Interval	P-Value	Heterogeneity (I^2^)	Certainty of Evidence (GRADE)
Intraoperative Blood Loss (WMD)	-215.48	(-367.58, -63.37)	P < 0.001	97%	Moderate
Transfusion Rate (RR)	0.40	(0.30, 0.53)	P < 0.001	46%	Moderate
Transfusion Rate (RD)	-0.22	(-0.67, 0.23)	0.33	98%	Moderate
Operation Time (WMD)	-15.82	(-53.35, 21.71)	0.41	97%	Moderate

### Intraoperative blood loss

**Fig 4:**
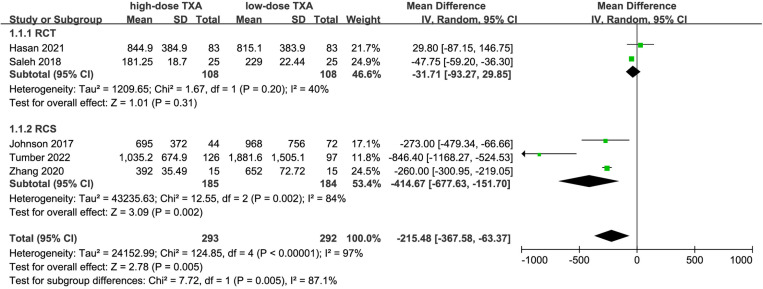
Forest plot of the effect of high-dose TXA on intraoperative blood loss.

**Fig 5:**
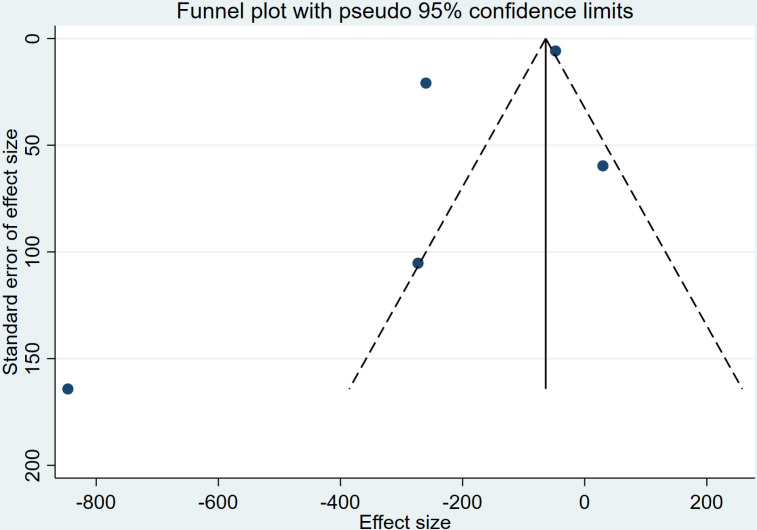
Funnel plots for intraoperative blood loss.

IBL data were extracted from 5 studies [14,19,20,22,23] involving 585 patients for comparing IBL between high-dose TXA and low-dose TXA. A pooled analysis revealed that high-dose TXA significantly reduced IBL, with a combined effect size of [WMD = -215.48, 95% CI (-367.58, -63.37), P <  0.001, Fig 4]. Due to the high heterogeneity among studies (I^2^ =  97%), a random-effects model was used for the final analysis. Subgroup analysis revealed that high-dose TXA significantly reduced IBL in RCS, while no significant difference was observed in RCT. Thus, based primarily on RCT results, there was no significant difference in IBL between high and low-dose TXA. Additionally, the funnel plot showed asymmetric dispersion of points, suggesting the presence of publication bias (Fig 5). According to the GRADE assessment, the quality of evidence was rated as moderate.

### Transfusion rate

**Fig 6:**
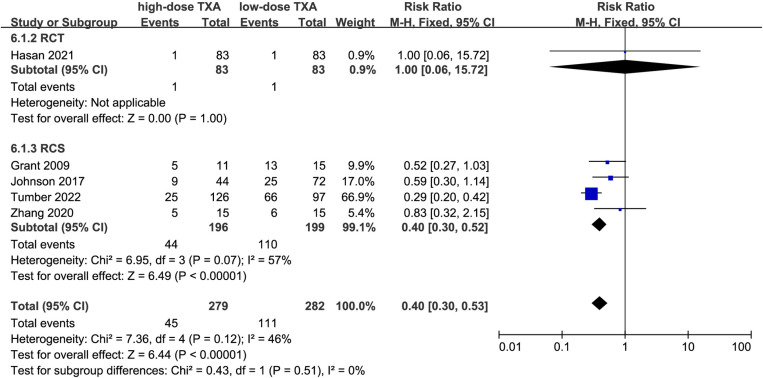
Forest plot of the effect of high-dose TXA on transfusion rate.

A systematic review of five studies [14,19,21–23], which collectively included 561 patients, was conducted to assess the impact of high-dose TXA on transfusion rates during spinal surgeries. The meta-analysis demonstrated a significant reduction in the need for transfusions [RR = 0.40,95% CI (0.30, 0.53), P <  0.001, Fig 6]. Due to the heterogeneity among the studies (I^2^ = 46%), a fixed-effects model was used to calculate the RR. The RD was -0.22, 95% CI (-0.67, 0.23), P = 0.33. Given the heterogeneity among the studies (I^2^ = 98%), a random-effects model was used to calculate the RD. Subgroup analysis revealed that high-dose TXA significantly reduces the transfusion rate in RCS, whereas no significant difference was observed in RCT. Therefore, based primarily on the results of RCTs, there is no significant difference in transfusion rates between high and low doses of TXA. The quality of the evidence was assessed as low.

### Operation time and thromboembolic events

**Fig 7:**
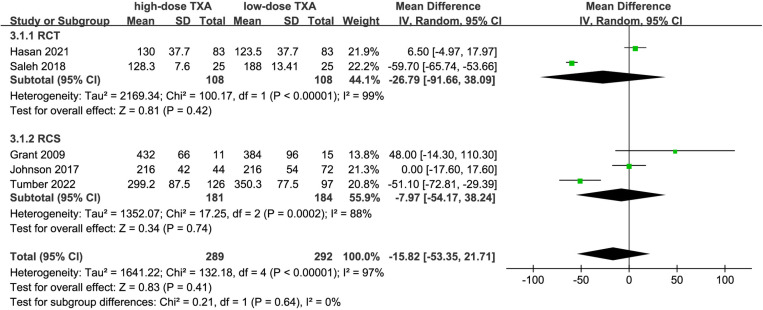
Forest plot of the effect of high-dose TXA on operation time.

Five studies [14,19–22] including 581 patients were used to evaluate operative time. The results showed no reduction in operative time with the use of high-dose TXA [WMD =  -15.82, 95% CI (-53.35, 21.71), P =  0.41, Fig 7]. Inter-study heterogeneity was high (I^2^ =  97%), so a random-effects model was used to analyze the final results. In addition, all pooled studies showed no thrombotic events. The quality of evidence was rated as moderate.

## Discussion

This meta-analysis aims to investigate the efficacy of TXA in the surgical treatment of AIS, with a particular focus on comparing the effects of high versus low doses of TXA. Unlike our previous research [24], which primarily focused on high-dose TXA versus placebo in AIS, this study compares high-dose TXA with low-dose TXA. Through a comprehensive analysis of six studies, including two RCTs and four RCSs, we found that, compared to low-dose TXA, high-dose TXA may not have a significant difference in terms of IBL, transfusion rates, and surgical time. This study represents the first systematic comparison of high and low doses of TXA in the context of AIS, whereas previous research primarily focused on comparisons between TXA and placebo groups.

In contrast to the findings of Raman et al. [25] in adult spinal deformity surgery, their study demonstrated that high-dose TXA (loading dose: 30-50 mg/kg, maintenance dose: 1-5 mg/kg/h) was more effective in reducing bleeding and transfusion requirements compared to low-dose TXA (loading dose: 10-20 mg/kg, maintenance dose: 1-2 mg/kg/h). However, our results indicate that there is no significant difference in IBL and transfusion rates between high and low-dose TXA. Subgroup analysis showed that high-dose TXA significantly reduced IBL and transfusion rates in RCS, while no significant difference was observed in RCTs. It is noteworthy that this outcome may be influenced by the limited number of RCTs included (two for IBL and only one for transfusion rates). Previous research by Zhong et al [26]. indicated that, compared to a placebo, TXA could reduce the duration of surgery, IBL, and transfusion rates in surgeries for AIS. However, our study showed that increasing the TXA dose from low to high did not further shorten the operation time. This is also consistent with the primary use of TXA in AIS surgery, which is to reduce bleeding and the need for transfusions.

TXA is an antifibrinolytic agent that reduces bleeding by inhibiting fibrinolysis. According to existing research, a low dose of TXA (10 mg/kg loading dose with a maintenance infusion of 1 mg/kg/h) can achieve a plasma concentration of 10 μg/ml, resulting in approximately 80% inhibition of fibrinolysis. To achieve near-complete fibrinolytic inhibition (98%), the plasma concentration of TXA needs to reach 100 μg/ml [27]. Additionally, high doses of TXA not only have a greater capacity to inhibit fibrinolysis but also possess anti-inflammatory properties, capable of suppressing plasmin-induced platelet activation [28]. However, it is important to note that high-dose TXA may increase the risk of thrombosis, and some studies have shown that TXA use is associated with thrombus formation [29,30]. In the field of cardiac surgery, the relationship between TXA and the risk of seizures is dose-dependent, with high doses of TXA increasing the risk of seizures [31–33].

Despite these potential risks, the use of high-dose TXA in this study did not result in an increased incidence of thromboembolic events, which is consistent with previous research on the use of TXA in spinal surgery [34]. This may be related to study design, as many studies exclude subjects with liver, kidney, and coagulation dysfunctions. Therefore, we cannot completely rule out the possibility that high-dose TXA may increase thrombotic risk in specific patient populations. In the field of cardiac surgery, studies on the pharmacokinetics of TXA have been conducted, leading to the development of corresponding dosing strategies [35,36]. However, in the field of spinal surgery, particularly for patients with AIS, there is still a lack of sufficient research. Spinal surgery often involves prolonged surgical procedures and anesthesia, so when using TXA in such operations, clinicians must balance its benefits in reducing blood loss against potential risks. This balance becomes especially crucial when the advantages of high-dose TXA over low-dose TXA in reducing blood loss have not yet been clearly established.

In summary, although the optimal dosage of TXA in the treatment of AIS remains unclear, our findings suggest that there may be no significant differences in IBL, transfusion rates, or operative time. Due to the inconsistency between the conclusions drawn from RCTs and RCSs, we tend to rely more on the results of RCTs. However, the number of included RCTs is limited (only two), and the small sample size makes it difficult to provide robust causal inferences. The differences between the results of RCTs and RCSs may be attributed to variations in study design. Therefore, these findings should be interpreted with caution. Additionally, future research should focus on conducting larger-scale, high-quality RCTs with standardized dosing regimens and extended follow-up periods. Simultaneously, efforts should be made to standardize the definitions of “high-dose” and “low-dose” TXA to enhance the comparability of study results and their applicability in clinical practice.

This meta-analysis also has several limitations. First, the number of studies included is limited, comprising only 2 RCTs and 4 RCSs. Furthermore, many of the studies had small sample sizes, which may affect the accuracy and generalizability of the results. Thus, while our analysis provides valuable insights, the findings must be approached with caution. Second, the specific doses of high and low TXA used in the included studies were not standardized, and the methods of perioperative blood loss assessment differ, which could impact the comparability and interpretation of the findings. Lastly, the follow-up duration concerning the safety of TXA in most studies was short, not allowing for a comprehensive assessment of long-term safety.

## Conclusion

In conclusion, high-dose TXA does not appear to offer significant advantages in reducing IBL, transfusion rates, or surgical time. Additionally, to more robustly establish the effectiveness and safety profile of high-dose TXA in spinal fusion procedures for individuals with AIS, further investigation through high-quality RCTs is imperative.

## Supporting information

S1 Table PRISMA 2020 checklist. (DOCX)

S2 Table Search strategy for PubMed. (DOCX)

S3 Table List of screened studies with inclusion status and reasons for exclusion. (DOCX)

S4 Table Data extraction table for systematic review and meta-analysis. (DOCX)
