# Supplementary material for: Comparative efficacy and safety of high-dose versus low-dose tranexamic acid in adolescent idiopathic scoliosis: A systematic review and meta-analysis
Source: PLoS One. 2025 Apr 1;20(4):e0320391. doi: 10.1371/journal.pone.0320391 (PMC11960895; doi:10.1371/journal.pone.0320391)
Supplement: S2 Table — (DOCX) [file pone.0320391.s002.docx]

Search strategy for PubMed.

| Number | Search terms |
| --- | --- |
| 1 | "tranexamic acid"[All Fields] |
| 2 | "TXA"[All Fields] |
| 3 | 1 OR 2 |
| 4 | "adolescent idiopathic scoliosis"[All Fields] |
| 5 | "AIS"[All Fields] |
| 6 | 4 OR 5 |
| 7 | 3 AND 6 |
