# Supplementary material for: Comparative efficacy and safety of high-dose versus low-dose tranexamic acid in adolescent idiopathic scoliosis: A systematic review and meta-analysis
Source: PLoS One. 2025 Apr 1;20(4):e0320391. doi: 10.1371/journal.pone.0320391 (PMC11960895; doi:10.1371/journal.pone.0320391)
Supplement: S3 Table — (DOCX) [file pone.0320391.s003.docx]

**List of Screened Studies with Inclusion Status and Reasons for Exclusion**

| **Reference** | **Included (Yes/No)** | **Reason for Exclusion** |
| --- | --- | --- |
| [No author listed],2017(1) | No | Irrelevant article |
| Abcejo,2017(2) | No | Irrelevant article |
| Abousamra,2019(3) | No | Irrelevant article |
| Acheampong,2012(4) | No | Irrelevant article |
| Actrn,2017(5) | No | Irrelevant article |
| Akosman,2023(6) | No | Irrelevant article |
| Alhammoud,2022(7) | No | Irrelevant article |
| Amin,2016(8) | No | Irrelevant article |
| Baidwan,2023(9) | No | Irrelevant article |
| Bassett,2021(10) | No | Irrelevant article |
| Battaglia,2018(11) | No | Irrelevant article |
| Benjamin,2024(12) | No | Irrelevant article |
| Berney,2015(13) | No | Lack of comparison between high-dose and low-dose tranexamic acid |
| Bess,2006(14) | No | Review |
| Boenigk,2011(15) | No | Irrelevant article |
| Boniello,2016(16) | No | Irrelevant article |
| Borden,2016(17) | No | Irrelevant article |
| Borgman,2014(18) | No | Irrelevant article |
| Bosch,2016(19) | No | Irrelevant article |
| Bosch,2019(20) | No | Lack of comparison between high-dose and low-dose tranexamic acid |
| Bourget-Murray,2019(21) | No | Irrelevant article |
| Bovonratwet,2020(22) | No | Irrelevant article |
| Bridwell,2014(23) | No | Irrelevant article |
| Cao,2022(24) | No | Irrelevant article |
| Chen,2015(25) | No | Irrelevant article |
| Chen,2020(26) | No | Irrelevant article |
| ChiCtr,2018(27) | No | Irrelevant article |
| ChiCtr,2019(28) | No | Irrelevant article |
| ChiCtr,2022(29) | No | Irrelevant article |
| ChiCtr,2023(30) | No | Irrelevant article |
| Chiu,2016(31) | No | Irrelevant article |
| Chung,2022(32) | No | Irrelevant article |
| Chung,2022(33) | No | Irrelevant article |
| Clark,2020(34) | No | Irrelevant article |
| Clark,2018(35) | No | Case Report |
| Contartese,2023(36) | No | Irrelevant article |
| Cooley,2020(37) | No | Irrelevant article |
| Croft,2015(38) | No | Irrelevant article |
| Das,2020(39) | No | Irrelevant article |
| David,2023(40) | No | Irrelevant article |
| Davis,2021(41) | No | Irrelevant article |
| DeVries,2020(42) | No | Irrelevant article |
| Dong,2021(43) | No | Lack of comparison between high-dose and low-dose tranexamic acid |
| Douglas,2020(44) | No | Irrelevant article |
| Durbin,(45) | No | Irrelevant article |
| Eaker,2022(46) | No | Irrelevant article |
| Eastridge,2012(47) | No | Irrelevant article |
| Eckert,2014(48) | No | Irrelevant article |
| Eidelson,2018(49) | No | Irrelevant article |
| En,2014(50) | No | Irrelevant article |
| Epstein,2015(51) | No | Irrelevant article |
| Epstein,2016(52) | No | Irrelevant article |
| Etrusco,2023(53) | No | Irrelevant article |
| Ezhevskaya,2018(54) | No | Irrelevant article |
| Fletcher,2022(55) | No | Irrelevant article |
| Fuentes Caparros,2017(56) | No | Irrelevant article |
| Gadiya,2021(57) | No | Irrelevant article |
| Galán-Olleros,2022(58) | No | Irrelevant article |
| Gatam,2019(59) | No | Irrelevant article |
| George,2021(60) | No | Lack of comparison between high-dose and low-dose tranexamic acid |
| Gianesello,2018(61) | No | Irrelevant article |
| Goobie,2018(62) | No | Irrelevant article |
| Goobie,2017(63) | No | Irrelevant article |
| Goobie,2017(64) | No | Irrelevant article |
| Goobie,2018(65) | No | Lack of comparison between high-dose and low-dose tranexamic acid |
| Gozubuyuk,2022(66) | No | Irrelevant article |
| Grana,2023(67) | No | Irrelevant article |
| Grana,2023(68) | No | Irrelevant article |
| Grant,2009(69) | Yes | - |
| Haleem,2022(70) | No | Irrelevant article |
| Halpern,2021(71) | No | Lack of comparison between high-dose and low-dose tranexamic acid |
| Hao,2015(72) | No | Irrelevant article |
| Hasan,2017(73) | No | Irrelevant article |
| Hasan,2021(74) | Yes | - |
| Hegeduš,2017(75) | No | Irrelevant article |
| Helenius,2016(76) | No | Irrelevant article |
| Helenius,2022(77) | No | Irrelevant article |
| Helsloot,2023(78) | No | Irrelevant article |
| Hengartner,2023(79) | No | Irrelevant article |
| Hengartner,2023(80) | No | Irrelevant article |
| Hideshima,2021(81) | No | Lack of comparison between high-dose and low-dose tranexamic acid |
| Hoefer,2024(82) | No | Irrelevant article |
| Hoelscher,2011(83) | No | Irrelevant article |
| Immel-Sehr,2007(84) | No | Irrelevant article |
| Ivasyk,2022(85) | No | Irrelevant article |
| Johnson,2017(86) | Yes | - |
| Jones,2017(87) | No | Lack of comparison between high-dose and low-dose tranexamic acid |
| Julien-Marsollier,2020(88) | No | Irrelevant article |
| Julien-Marsollier,2023(89) | No | Irrelevant article |
| Kanakaris,2023(90) | No | Irrelevant article |
| Keon,2018(91) | No | Irrelevant article |
| Kolz,2022(92) | No | Irrelevant article |
| Lee,2017(93) | No | Irrelevant article |
| Liu,2024(94) | No | Irrelevant article |
| Liu,2024(95) | No | Irrelevant article |
| Lonner,2018(96) | No | Irrelevant article |
| Lykissas,2013(97) | No | Lack of comparison between high-dose and low-dose tranexamic acid |
| Mahmoud,2021(98) | No | Irrelevant article |
| Mallepally,2023(99) | No | Irrelevant article |
| Mallet,2022(100) | No | Irrelevant article |
| Mari,2022(101) | No | Irrelevant article |
| McLeod,2013(102) | No | Irrelevant article |
| McLeod,2015(103) | No | Irrelevant article |
| McNicol,2016(104) | No | Meta analysis |
| Meyer,2017(105) | No | Irrelevant article |
| Mihas,2021(106) | No | Irrelevant article |
| Miller,2018(107) | No | Irrelevant article |
| Minhas,2015(108) | No | Irrelevant article |
| Mirzashahi,2020(109) | No | Irrelevant article |
| Morte,2019(110) | No | Irrelevant article |
| Nagabhushan,2018(111) | No | Irrelevant article |
| Nct,2009(112) | No | Irrelevant article |
| Nct,2013(113) | No | Irrelevant article |
| Nct,2018(114) | No | Irrelevant article |
| Nct,2022(115) | No | Irrelevant article |
| Newall,2021(116) | No | Irrelevant article |
| Ng,2015(117) | No | Lack of comparison between high-dose and low-dose tranexamic acid |
| Niemeyer,2022(118) | No | Irrelevant article |
| Nugent,2016(119) | No | Irrelevant article |
| O'Donnell,2017(120) | No | Irrelevant article |
| O'Donoghue,2020(121) | No | Irrelevant article |
| Oetgen,2017(122) | No | Review |
| Ohashi,2017(123) | No | Lack of comparison between high-dose and low-dose tranexamic acid |
| Ohrt-Nissen,2017(124) | No | Irrelevant article |
| Ohrt-Nissen,2017(125) | No | Irrelevant article |
| Oliveira,2018(126) | No | Irrelevant article |
| Owen,2021(127) | No | Irrelevant article |
| Pactr,2018(128) | No | Irrelevant article |
| Palmer,2010(129) | No | Irrelevant article |
| Pennington,2021(130) | No | Irrelevant article |
| Pennington,2020(131) | No | Irrelevant article |
| Peters,2013(132) | No | Irrelevant article |
| Pico,2022(133) | No | Irrelevant article |
| Prasad,2024(134) | No | Irrelevant article |
| Qureshi,2017(135) | No | Review |
| Rajanigandha,2023(136) | No | Irrelevant article |
| Ramgren,2017(135) | No | Irrelevant article |
| Saleh,2018(137) | Yes | - |
| Sarwahi,2016(138) | No | Irrelevant article |
| Sarwahi,2024(139) | No | Irrelevant article |
| Shackelford,2021(140) | No | Irrelevant article |
| Shchederkina,2022(141) | No | Irrelevant article |
| Shrestha,2021(142) | No | Meta analysis |
| Soini,2023(143) | No | Irrelevant article |
| Soliman,2019(144) | No | Irrelevant article |
| Song,2021(145) | No | Lack of comparison between high-dose and low-dose tranexamic acid |
| Stein,2019(146) | No | Irrelevant article |
| Sui,2016(147) | No | Lack of comparison between high-dose and low-dose tranexamic acid |
| Süzer,2023(148) | No | Irrelevant article |
| Tan,2018(149) | No | Irrelevant article |
| Tan,2023(150) | No | Irrelevant article |
| Thompson,2005(151) | No | Irrelevant article |
| Toombs,2018(152) | No | Irrelevant article |
| Tumber,2022(153) | Yes | - |
| Uehara,2019(154) | No | Irrelevant article |
| Urbanski,2023(155) | No | Irrelevant article |
| Van Haren,2014(156) | No | Irrelevant article |
| van Popta,2014(157) | No | Irrelevant article |
| van Wessem,2022(158) | No | Irrelevant article |
| Vasan,2021(159) | No | Irrelevant article |
| Verma,2014(160) | No | Lack of comparison between high-dose and low-dose tranexamic acid |
| Verma,2010(161) | No | Lack of comparison between high-dose and low-dose tranexamic acid |
| Von kier,2021(162) | No | Irrelevant article |
| Wada,2021(163) | No | Irrelevant article |
| Wagala,2021(164) | No | Irrelevant article |
| Wahlquist,2017(165) | No | Irrelevant article |
| Wang,2021(166) | No | Irrelevant article |
| Wang,2024(167) | No | Irrelevant article |
| Wirtz,2018(168) | No | Irrelevant article |
| Wolff,2019(169) | No | Irrelevant article |
| Wu,2023(170) | No | Irrelevant article |
| Xie,2015(171) | No | Irrelevant article |
| Xu,2012(172) | No | Lack of comparison between high-dose and low-dose tranexamic acid |
| Yagi,2012(173) | No | Lack of comparison between high-dose and low-dose tranexamic acid |
| Yan,2021(174) | No | Irrelevant article |
| Yang,2021(175) | No | Irrelevant article |
| Ye,2023(176) | No | Irrelevant article |
| Yunus,2019(177) | No | Irrelevant article |
| Zhang,2020(178) | Yes | - |
| Zhang,2021(179) | No | Lack of comparison between high-dose and low-dose tranexamic acid |
| Zhang,2023(180) | No | Irrelevant article |
| Zhong,2019(181) | No | Meta analysis |
| Zielinski,2015(182) | No | Irrelevant article |

**Reference**：

1. SABM Abstracts. Anesthesia and Analgesia. 2017;125(3).

2. Abcejo AS, Diaz Soto J, Castoro C, Armour S, Long TR. Profound obstructive hypotension from prone positioning documented by transesophageal echocardiography in a patient with scoliosis: A case report. A and A Case Reports. 2017;9(3):87-9.

3. Abousamra O, Sponseller PD, Lonner BS, Shah SA, Marks MC, Cahill PJ, et al. Thoracic Lordosis, Especially in Males, Increases Blood Loss in Adolescent Idiopathic Scoliosis. J Pediatr Orthop. 2019;39(3):e201-e4.

4. Acheampong P, Ford GA. Pharmacokinetics of alteplase in the treatment of ischaemic stroke. Expert Opinion on Drug Metabolism and Toxicology. 2012;8(2):271-81.

5. Actrn. Tranexamic Acid in Paediatric Scoliosis Surgery (TRIPSS). <https://trialsearchwhoint/Trial2aspx?TrialID=ACTRN12617000663358>. 2017.

6. Akosman I, Lovecchio F, Fourman M, Sarmiento M, Lyons K, Memtsoudis S, et al. Is High-Dose Tranexamic Safe in Spine Surgery? A Systematic Review and Meta-Analysis. GLOBAL SPINE JOURNAL. 2023;13(7):2085-95.

7. Alhammoud A, Alborno Y, Baco AM, Othman YA, Ogura Y, Steinhaus M, et al. Minimally Invasive Scoliosis Surgery Is a Feasible Option for Management of Idiopathic Scoliosis and Has Equivalent Outcomes to Open Surgery: A Meta-Analysis. GLOBAL SPINE JOURNAL. 2022;12(3):483-92.

8. Amin S, Siafaka C, Murugan A, Mallick A. Paediatric arterial ischaemic stroke secondary to internal carotid artery dissection following chiropractic activation therapy. International Journal of Stroke. 2016;11:144.

9. Baidwan NK, Schauer SG, Dixon JM, Bhaumik S, April MD, April MD, et al. Tranexamic Acid Improves Survival in the Setting of Severe Head Injury in Combat Casualties. Medical journal (Fort Sam Houston, Tex). 2023(Per 23-1/2/3):34-40.

10. Bassett W, Caruso C, Adolfsen S, McPartland T, AndrewBowe J, Tuason D. A two-surgeon approach improves performance for young surgeons in adolescent idiopathic scoliosis. Orthopedics. 2021;44(3):E347-E52.

11. Battaglia S, Vega M, Grifi M. BLOOD LOSS AND TRANSFUSION MANAGEMENT IN COMPLEX MULTI-LEVEL SPINE SURGERY IN EUROPE. Value in Health. 2018;21:S343-S4.

12. Benjamin AJ, Polcari AM. TXA does not affect levels of TBI-related biomarkers in blunt TBI with ICH: A secondary analysis of the prehospital TXA for TBI trial. Journal of Trauma and Acute Care Surgery. 2024;96(1):94-100.

13. Berney MJ, Dawson PH, Phillips M, Lui DF, Connolly P. Eliminating the use of allogeneic blood products in adolescent idiopathic scoliosis surgery. Eur J Orthop Surg Traumatol. 2015;25 Suppl 1:S219-23.

14. Bess RS, Lenke LG. Blood Loss Minimization and Blood Salvage Techniques for Complex Spinal Surgery. Neurosurgery Clinics of North America. 2006;17(3):227-34.

15. Boenigk K, Verma K, Hoelscher C, Huncke KT, Lonner B, Errico T. The efficacy of antifibrinoly tics at reducing blood loss in major spine surgery: a prospective randomized comparison of tranexamic acid, aminocaproic acid, and placebo. European journal of anaesthesiology. 2011;28:90.

16. Boniello AJ, Verma K, Peters A, Lonner BS, Errico T. Pre-operative autologous blood donation does not affect pre-incision hematocrit in adolescent idiopathic scoliosis patients. a retrospective cohort of a prospective randomized trial. International Journal of Spine Surgery. 2016;10(2016).

17. Borden TC, Bellaire LL, Fletcher ND. Improving perioperative care for adolescent idiopathic scoliosis patients: the impact of a multidisciplinary care approach. Journal of multidisciplinary healthcare. 2016;9:435-45.

18. Borgman M, Spinella P, Pidcoke H, Cap A, Cannon J. Tranexamic acid for pediatric trauma. Critical Care Medicine. 2014;42(12):A1607-A8.

19. Bosch P, Kenkre TS, Londino JA, Cassara A, Yang C, Waters JH. Coagulation Profile of Patients with Adolescent Idiopathic Scoliosis Undergoing Posterior Spinal Fusion. JOURNAL OF BONE AND JOINT SURGERY-AMERICAN VOLUME. 2016;98(20).

20. Bosch P, Kenkre TS, Soliman D, Londino JA, Novak NE. Comparison of the Coagulation Profile of Adolescent Idiopathic Scoliosis Patients Undergoing Posterior Spinal Fusion With and Without Tranexamic Acid. Spine Deform. 2019;7(6):910-6.

21. Bourget-Murray J, Brown GE, Peiro-Garcia A, Earp MA, Parsons DL, Ferri-de-Barros F. Quality, Safety, and Value of Innovating Classic Operative Techniques in Scoliosis Surgery: Intraoperative Traction and Navigated Sequential Drilling. Spine Deformity. 2019;7(4):588-95.

22. Bovonratwet P, Sheha ED, Ondeck NT, Malpani R, Smith BG, Grauer JN. Safety and Effectiveness of Antifibrinolytics in Posterior Scoliosis Surgery for Adolescent Idiopathic Scoliosis An Analysis of the NSQIP-Pediatric Database. CLINICAL SPINE SURGERY. 2020;33(1):E26-E32.

23. Bridwell KH, Anderson PA, Boden SD, Kim HJ, Vaccaro AR, Wang JC. What's new in spine surgery. Journal of Bone and Joint Surgery. 2014;96(12):1048-54.

24. Cao Z, Li Q, Guo J, Li Y, Wu J. Optimal administration strategies of tranexamic acid to minimize blood loss during spinal surgery: results of a Bayesian network meta-analysis. ANNALS OF MEDICINE. 2022;54(1):2053-63.

25. Chen H, Kan S. Clinical Study on the Effect of Tranexamic Acid in Reducing Blood Loss in Posterior Fusion Surgery for Adolescent Idiopathic Scoliosis. Tianjin Medical Journal. 2015;43(09):1044-6.

26. Chen W, Shen J, Zhang Y, Hu A, Liang J, Ma L, et al. A randomised controlled trial of fibrinogen concentrate during scoliosis surgery. Anaesthesia. 2020;75(11):1476-81.

27. ChiCtr. Application of tranexamic acid enhanced recovery after correction surgery for spinal deformity to reduce postoperative hemorrhage: a prospective randomized controlled trial. <https://trialsearchwhoint/Trial2aspx?TrialID=ChiCTR1800017565>. 2018.

28. ChiCtr. Topic injection of tranexamic acid via a drain plus drain-clamping to reduce blood loss in adolescent idiopathic scoliosis surgery: a randomized controlled trial. <https://trialsearchwhoint/Trial2aspx?TrialID=ChiCTR1900024177>. 2019.

29. ChiCtr. Study on the safety and efficacy of tranexamic acid on bleeding in spinal surgery. <https://trialsearchwhoint/Trial2aspx?TrialID=ChiCTR2200064659>. 2022.

30. ChiCtr. Study on safety and efficacy of tranexamic acid combined with gum in spinal surgery. <https://trialsearchwhoint/Trial2aspx?TrialID=ChiCTR2300067944>. 2023.

31. Chiu CK, Chan CYW, Aziz I, Hasan MS, Kwan MK. Assessment of Intraoperative Blood Loss at Different Surgical Stages During Posterior Spinal Fusion Surgery in the Treatment of Adolescent Idiopathic Scoliosis. SPINE. 2016;41(9):E566-E73.

32. Chung WH, Lee YJ, Chiu CK, Hasan MS, Chan CYW, Kwan MK. Severe Lenke 1 and 2 adolescent idiopathic scoliosis had poorer perioperative outcome, higher complication rate, longer fusion and higher operative cost compared to non-severe scoliosis. European Spine Journal. 2022;31(4):1051-9.

33. Chung WH, Mihara Y, Chiu CK, Hasan MS, Chan CYW, Kwan MK. Factors Affecting Operation Duration in Posterior Spinal Fusion (PSF) Using Dual Attending Surgeon Strategy Among Lenke 1 and 2 Adolescent Idiopathic Scoliosis (AIS) Patients. Clinical Spine Surgery. 2022;35(1):18-23.

34. Clark JP, Diab M. Neurophysiologic detection of spinal cord ischemia during anterior vertebral tethering. Spine. 2020;45(24):E1703-E6.

35. Clark N, Morris S, Sargant N. Comment on Chiem J, Ivanova I, Jimenez N. Anaphylactic reaction to tranexamic acid in an adolescent undergoing posterior spinal fusion. Paediatric Anaesthesia. 2018;28(2):184-5.

36. Contartese D, Salamanna F, Brogini S, Martikos K, Griffoni C, Ricci A, et al. Fast-track protocols for patients undergoing spine surgery: a systematic review. BMC MUSCULOSKELETAL DISORDERS. 2023;24(1).

37. Cooley R, Zhao H, Coote S, Easton D, Langenberg F, Smith K, et al. Mobile stroke units facilitate management of intracranial hemorrhage. International Journal of Stroke. 2020;15(1 SUPPL):530.

38. Croft LD, Pottinger JM, Chiang H-Y, Ziebold CS, Weinstein SL, Herwaldt LA. Risk Factors for Surgical Site Infections After Pediatric Spine Operations. SPINE. 2015;40(2):E112-E9.

39. Das S, Mondal GP, Bhattacharya R, Ghosh KC, Das S, Pattem HK, et al. Predictors of Postthrombolysis Outcome and Symptomatic Postthrombolysis Hemorrhage following Intravenous Thrombolysis with Alteplase for Acute Ischemic Stroke. Journal of Neurosciences in Rural Practice. 2020;11(2):315-24.

40. David J-S, James A, Orion M, Selves A, Bonnet M, Glasman P, et al. Thromboelastometry-guided haemostatic resuscitation in severely injured patients: a propensity score-matched study. CRITICAL CARE. 2023;27(1).

41. Davis S, Donnan G, Cooley R, Zhao H, Campbell B, Churilov L, et al. Prehospital strategies for brain hemorrhage management. International Journal of Stroke. 2021;16(2 SUPPL):3.

42. DeVries Z, Barrowman N, Smit K, Mervitz D, Moroz P, Tice A, et al. Is it feasible to implement a rapid recovery pathway for adolescent idiopathic scoliosis patients undergoing posterior spinal instrumentation and fusion in a single-payer universal health care system? Spine Deformity. 2020;8(6):1223-9.

43. Dong Y, Liang J, Tong B, Shen J, Zhao H, Li Q. Combined topical and intravenous administration of tranexamic acid further reduces postoperative blood loss in adolescent idiopathic scoliosis patients undergoing spinal fusion surgery: a randomized controlled trial. BMC musculoskeletal disorders. 2021;22(1):663.

44. Douglas DR, Luoma V, Reddy U. Acute management of ischaemic stroke. Anaesthesia and Intensive Care Medicine. 2020;21(1):1-7.

45. Durbin S, Brito A, Johnson A, Cotton B, Rowell S, Schreiber MA. Association of Fibrinolysis Phenotype with Patient Outcomes following Traumatic Brain Injury. The journal of trauma and acute care surgery. 2024;96(3):482‐6.

46. Eaker L, Selverian SR, Hodo LN, Gal J, Gangadharan S, Meyers J, et al. Post-operative tranexamic acid decreases chest tube drainage following vertebral body tethering surgery for scoliosis correction. Spine Deform. 2022;10(4):811-6.

47. Eastridge BJ, Mabry RL, Seguin P, Cantrell J, Tops T, Uribe P, et al. Death on the battlefield (2001-2011): Implications for the future of combat casualty care. JOURNAL OF TRAUMA AND ACUTE CARE SURGERY. 2012;73:S431-S7.

48. Eckert MJ, Wertin TM, Tyner SD, Nelson DW, Izenberg S, Martin MJ. Tranexamic acid administration to pediatric trauma patients in a combat setting: the pediatric trauma and tranexamic acid study (PED-TRAX). J Trauma Acute Care Surg. 2014;77(6):852-8; discussion 8.

49. Eidelson S, Karcutskie C, Mulder M, Meizoso J, Padiadpu A, Rattan R, et al. Is tranexamic acid associated with infection in critically injured trauma patients? Critical Care Medicine. 2018;46:787.

50. En X. Efficacy of reptilase, tranexamic acid or both combined in adolescent idiopathic scoliosis surgery: a prospective, randomized, double-blind, placebo-controlled study. Spine journal. 2014;14(11):S35.

51. Epstein DS, Mitra B, Cameron PA, Fitzgerald M, Rosenfeld JV. Acute traumatic coagulopathy in the setting of isolated traumatic brain injury: Definition, incidence and outcomes. BRITISH JOURNAL OF NEUROSURGERY. 2015;29(1):118-22.

52. Epstein DS, Mitra B, Cameron PA, Fitzgerald M, Rosenfeld JV. Normalization of coagulopathy is associated with improved outcome after isolated traumatic brain injury. J Clin Neurosci. 2016;29:64-9.

53. Etrusco A, Barra F, Chiantera V, Ferrero S, Bogliolo S, Evangelisti G, et al. Current Medical Therapy for Adenomyosis: From Bench to Bedside. Drugs. 2023;83(17):1595-611.

54. Ezhevskaya AA, Prusakova ZB, Zagrekov VI, Sosnin AV, Milenovic M. Efficacy assessment of epidural blockade and tranexamic acid application in idiopathic scoliosis surgery. Sovremennye tehnologii v medicine. 2018;10(4):164‐72.

55. Fletcher ND, Gilbertson LE, Bruce RW, Lewis M, Lam H, Austin TM. Blood loss estimation during posterior spinal fusion for adolescent idiopathic scoliosis. Spine Deformity. 2022;10(3):581-8.

56. Fuentes Caparros S, Echerei Z, Gavilén Martínez IA, Marín Lujén MA, Rodríguez De Tembleque Aguilar F, Gonzélez Barrios I. Bleeding and transfusion patterns in idiopathic scoliosis with low-dose tranexamic acid use: Prospective study. European Spine Journal. 2017;26(10):2703.

57. Gadiya AD, Koch JEJ, Patel MS, Shafafy M, Grevitt MP, Quraishi NA. Enhanced recovery after surgery (ERAS) in adolescent idiopathic scoliosis (AIS): a meta-analysis and systematic review. Spine Deformity. 2021;9(4):893-904.

58. Galán-Olleros M, Egea-Gámez RM, González-Díaz R. ISCHEMIC STROKE FOLLOWING IDIOPATHIC SCOLIOSIS SURGERY IN PEDIATRIC PATIENTS. European Spine Journal. 2022;31(2):548-9.

59. Gatam L, Mahadhipta H, Phedy, Gatam AR, Marsetio AF, Antoro A. Aphasia after spinal deformity correction surgery in adolescent idiopathic scoliosis: A case report. Journal of Global Pharma Technology. 2019;11(4):115-22.

60. George S, Ramchandran S, Mihas A, George K, Mansour A, Errico T. Topical tranexemic acid reduces intra-operative blood loss and transfusion requirements in spinal deformity correction in patients with adolescent idiopathic scoliosis. Spine Deform. 2021;9(5):1387-93.

61. Gianesello L, Coppo M, Attanasio M, Paniccia R, Boccacini A, De Gaudio AR. Evaluation of tranexamic acid efficacy in decreasing operative blood loss during adolescent idiopathic scoliosis surgery using rotem® monitoring. Blood transfusion. 2018;16:s525.

62. Goobie S. Tranexamic acid inhibits plasminogen in adolescent scoliosis surgery: a new approach to defining efficacy and antifibriolytic action. Anesthesia and analgesia. 2018;127(3):64‐5.

63. Goobie SM, Hresko MT, McCann ME, Brustowicz R, Glotzbecker M, Hedequist D, et al. Dose-response relationship of tranexamic acid in pediatric scoliosis surgery. Anesthesia and analgesia. 2017;125(3):1.

64. Goobie SM, Zurakowski, Brustowicz R, Glotzbecker M, Hedequist D, Sethna N, et al. Tranexamic acid decreases the rate of blood loss in adolescent idiopathic scoliosis surgery. Anesthesia and analgesia. 2017;125(3):7‐8.

65. Goobie SM, Zurakowski D, Glotzbecker MP, McCann ME, Hedequist D, Brustowicz RM, et al. Tranexamic Acid Is Efficacious at Decreasing the Rate of Blood Loss in Adolescent Scoliosis Surgery: a Randomized Placebo-Controlled Trial. Journal of bone and joint surgery American volume. 2018;100(23):2024‐32.

66. Gozubuyuk E, Aygun E, Basaran I, Canbolat N, Cavdaroglu B, Akgul T, et al. Effects of Changes in Body Temperature on Perioperative Bleeding in Adolescent Idiopathic Scoliosis Surgery. Therapeutic Hypothermia and Temperature Management. 2022;12(3):146-54.

67. Grana RM, Martins SCM, Mistro Neto S, Veiga IG, FrazÃOrosa A, Limal MC, et al. Coluna/Columna. 2023;22(3):e273367-e.

68. Grana RM, Martins SCM, Neto SM, Veiga IG, Frazaorosa A, Limal MC, et al. TRANEXAMIC ACID IN ADOLESCENT SCOLIOSIS SURGERY: A SYSTEMATIC REVIEW. Coluna/ Columna. 2023;22(3).

69. Grant JA, Howard J, Luntley J, Harder J, Aleissa S, Parsons D. Perioperative blood transfusion requirements in pediatric scoliosis surgery: the efficacy of tranexamic acid. J Pediatr Orthop. 2009;29(3):300-4.

70. Haleem S, Thimmaiah R, Nagrath N, Gowda D, Bhimarasetty C, Mehta JS. The impact of blood conservation techniques on transfusion requirements for posterior adolescent idiopathic scoliosis corrections: do we need a routine cross-match for the operation? Spine Deformity. 2022;10(3):589-93.

71. Halpern LM, Bronson WE, Kogan CJ. A New Low Dose of Tranexamic Acid for Decreasing the Rate of Blood Loss in Posterior Spinal Fusion for Adolescent Idiopathic Scoliosis. Journal of pediatric orthopedics. 2021;41(6):333‐7.

72. Hao D, Xie E. Efficacy and safety of prophylactic large dose of tranexamic acid in adolescent with idiopathic scoliosis surgery: a prospective, randomized, double-blind, placebo-controlled study. European spine journal. 2015;24(6):S702‐S3.

73. Hasan MS, Choe NC, Chan CYW, Chiu CK, Kwan MK. Effect of intraoperative autologous transfusion techniques on perioperative hemoglobin level in idiopathic scoliosis patients undergoing posterior spinal fusion: A prospective randomized trial. JOURNAL OF ORTHOPAEDIC SURGERY. 2017;25(2).

74. Hasan MS, Yunus SN, Ng CC, Chan CYW, Chiu CK, Kwan MK. Tranexamic Acid in Pediatric Scoliosis Surgery: a Prospective Randomized Trial Comparing High-dose and Low-dose Tranexamic Acid in Adolescent Idiopathic Scoliosis Undergoing Posterior Spinal Fusion Surgery. Spine. 2021;46(22):E1170‐E7.

75. Hegeduš I, Milić J, Ćosić A, Buljan K, Drenjančević I. Cerebrovascular reactivity in acute hyperoxia in patients with acute ischaemic stroke. Brain Inj. 2017;31(4):560-6.

76. Helenius I, Keskinen H, Syvänen J, Lukkarinen H, Mattila M, Välipakka J, et al. Gelatine matrix with human thrombin decreases blood loss in adolescents undergoing posterior spinal fusion for idiopathic scoliosis: a multicentre, randomised clinical trial. The bone & joint journal. 2016;98‐B(3):395‐401.

77. Helenius L, Gerdhem P, Ahonen M, Syvanen J, Jalkanen J, Charalampidis A, et al. Postoperative outcomes of pedicle screw instrumentation for adolescent idiopathic scoliosis with and without a subfascial wound drain: a multicentre randomized controlled trial. BONE & JOINT JOURNAL. 2022;104B(9):1067-72.

78. Helsloot D, Fitzgerald MC, Lefering R, Verelst S, Missant C. The first hour of trauma reception is critical for patients with major thoracic trauma: A retrospective analysis from the TraumaRegister DGU. European Journal of Anaesthesiology. 2023;40(11):865-73.

79. Hengartner AC, David WB, Reeves BC, Craft S, Boroumand S, Clappier M, et al. Effects of delayed ambulation following posterior spinal fusion for adolescent idiopathic scoliosis: a single institutional study. Spine Deformity. 2023;11(5):1127-36.

80. Hengartner AC, Havlik J, David WB, Reeves BC, Freedman IG, Sarkozy M, et al. Association Between Intravenous to Oral Opioid Transition Time and Length of Hospital Stay After Posterior Spinal Fusion for Adolescent Idiopathic Scoliosis. International Journal of Spine Surgery. 2023;17(3):468-76.

81. Hideshima T, Akazawa T, Iinuma M, Torii Y, Ueno J, Yoshida A, et al. Tranexamic Acid Reduces Total Blood Loss and the Amount of Stored Preoperative Autologous Blood Donation Needed for Adolescent Idiopathic Scoliosis Patients Undergoing Posterior Spinal Fusion. Cureus. 2021;13(6):e15488.

82. Hoefer LE, Benjamin AJ, Polcari AM, Schreiber MA, Zakrison TL, Rowell SE. TXA does not affect levels of TBI-related biomarkers in blunt TBI with ICH: a secondary analysis of the Prehospital TXA for TBI Trial. The journal of trauma and acute care surgery. 2024;96(1):94‐100.

83. Hoelscher C, Verma K, Errico T, Dryer J, Huncke T, Boenigk K, et al. Are anti-fibrinolytics effective at reducing peri-operative blood loss in adolescent idiopathic scoliosis? Spine journal. 2011;11(10):67S.

84. Immel-Sehr A. Finding individual solutions to the premenstrual syndrome. Pharmazeutische Zeitung. 2007;152(27):16-23.

85. Ivasyk I, Chatterjee A, Jordan C, Geiselmann MT, Chang PS, Kamel H, et al. Evaluation of the safety of tranexamic acid use in pediatric patients undergoing spinal fusion surgery: a retrospective comparative cohort study. BMC Musculoskelet Disord. 2022;23(1):651.

86. Johnson DJ, Johnson CC, Goobie SM, Nami N, Wetzler JA, Sponseller PD, et al. High-dose Versus Low-dose Tranexamic Acid to Reduce Transfusion Requirements in Pediatric Scoliosis Surgery. JOURNAL OF PEDIATRIC ORTHOPAEDICS. 2017;37(8):E552-E7.

87. Jones KE, Butler EK, Barrack T, Ledonio CT, Forte ML, Cohn CS, et al. Tranexamic Acid Reduced the Percent of Total Blood Volume Lost During Adolescent Idiopathic Scoliosis Surgery. Int J Spine Surg. 2017;11(4):27.

88. Julien-Marsollier F, Michelet D, Assaker R, Doval A, Louisy S, Madre C, et al. Enhanced recovery after surgical correction of adolescent idiopathic scoliosis. Paediatric Anaesthesia. 2020;30(10):1068-76.

89. Julien-Marsollier F, Penisson L, Happiette A, Ilharreborde B. Can hydroxyapatite charged collagen sponge help reduce perioperative blood loss in adolescent idiopathic scoliosis surgery? Preliminary results in 68 patients. EUROPEAN SPINE JOURNAL. 2023;32(3):883-8.

90. Kanakaris NK, Bouamra O, Lecky F, Giannoudis PV. Severe trauma with associated pelvic fractures: The impact of regional trauma networks on clinical outcome. INJURY-INTERNATIONAL JOURNAL OF THE CARE OF THE INJURED. 2023;54(6):1670-6.

91. Keon LJZ. Efficacy of Tranexamic Acid and Cell Salvage in Conserving Blood in Scoliosis Surgery2018 2018.

92. Kolz JM, Neal KM. Hidden blood loss in adolescent idiopathic scoliosis surgery. Orthopaedics and Traumatology: Surgery and Research. 2022;108(6).

93. Lee YAZ. Comparing the Effiacy of Tranexamic Acid and Aminocaproic Acid in Posterior Spinal Fusion for Adolescent Idiopathic Scoliosis2017 2017.

94. Liu DS, Farid AR, Linden GS, Cook D, Birch CM, Hresko MT, et al. Utility of postoperative laboratory testing after posterior spinal fusion for adolescent idiopathic scoliosis. SPINE DEFORMITY. 2024;12(2):375-81.

95. Liu X, Ma Z, Zhang X, Li S, An J, Luo Z. Research Progress of Long Non-coding RNA-ZFAS1 in Malignant Tumors. Cell Biochem Biophys. 2024.

96. Lonner BS, Ren Y, Asghar J, Shah SA, Samdani AF, Newton PO. Antifibrinolytic Therapy in Surgery for Adolescent Idiopathic Scoliosis Does the Level 1 Evidence Translate to Practice? Bull Hosp Jt Dis (2013). 2018;76(3):165-70.

97. Lykissas MG, Crawford AH, Chan G, Aronson LA, Al-Sayyad MJ. The effect of tranexamic acid in blood loss and transfusion volume in adolescent idiopathic scoliosis surgery: a single-surgeon experience. J Child Orthop. 2013;7(3):245-9.

98. Mahmoud L, Zullo A, Shu L, Yaghi S, Liberman AL, Lee V, et al. Association of tranexamic acid use with outcomes in alteplase-associated intracranial hemorrhage. Stroke. 2021;52(SUPPL 1).

99. Mallepally AR, Marathe N, Rustagi T, Mohapatra B, Mahanjan R, Das K. Management of AO-type C thoracolumbar fractures during COVID-19 pandemic using distractor device: a novel technique. British Journal of Neurosurgery. 2023;37(6):1928-35.

100. Mallet C, Meissburger V, Caseris M, Happiette A, Chinnappa J, Bonacorsi S, et al. Does the use of intrawound povidone-iodine irrigation and local vancomycin powder impact surgical site infection rate in adolescent idiopathic scoliosis surgery? European Spine Journal. 2022;31(11):3020-8.

101. Mari D, Biswas A. Fluid restriction and txa bundle during adolescent spinal surgery affects anemia and coagulopathy. Critical Care Medicine. 2022;50(1 SUPPL):320.

102. McLeod LM, French B, Flynn JM, Dormans JP, Keren R. Antifibrinolytic use and blood transfusions in pediatric scoliosis surgeries performed at US children's hospitals. Journal of Spinal Disorders and Techniques. 2013.

103. McLeod LM, French B, Flynn JM, Dormans JP, Keren R. Antifibrinolytic Use and Blood Transfusions in Pediatric Scoliosis Surgeries Performed at US Children's Hospitals. J Spinal Disord Tech. 2015;28(8):E460-6.

104. McNicol ED, Tzortzopoulou A, Schumann R, Carr DB, Kalra A. Antifibrinolytic agents for reducing blood loss in scoliosis surgery in children. Cochrane Database Syst Rev. 2016;9(9):Cd006883.

105. Meyer RM, Larkin MB, Szuflita NS, Neal CJ, Tomlin JM, Armonda RA, et al. Early venous thromboembolism chemoprophylaxis in combat-related penetrating brain injury. J Neurosurg. 2017;126(4):1047-55.

106. Mihas A, Ramchandran S, Rivera S, Mansour A, Asghar J, Shufflebarger H, et al. Safe and effective performance of pediatric spinal deformity surgery in patients unwilling to accept blood transfusion: a clinical study and review of literature. BMC Musculoskeletal Disorders. 2021;22(1).

107. Miller DJ, Cahill PJ, Janicki JA, Stephenson LP, Choi PD. What's New in Pediatric Orthopaedic Quality, Safety, and Value? A Systematic Review With Results of the 2016 POSNA Quality, Safety, and Value Initiative (QSVI) Challenge. JOURNAL OF PEDIATRIC ORTHOPAEDICS. 2018;38(10):E646-E51.

108. Minhas SV, Chow I, Bosco J, Otsuka NY. Assessing the Rates, Predictors, and Complications of Blood Transfusion Volume in Posterior Arthrodesis for Adolescent Idiopathic Scoliosis. SPINE. 2015;40(18):1422-30.

109. Mirzashahi B, Moosavi M, Rostami M. Outcome of posterior-only approach for severe rigid scoliosis: A retrospective report. International Journal of Spine Surgery. 2020;14(2):232-8.

110. Morte D, Lammers D, Bingham J, Kuckelman J, Eckert M, Martin M. Tranexamic acid administration following head trauma in a combat setting: Does tranexamic acid result in improved neurologic outcomes? J Trauma Acute Care Surg. 2019;87(1):125-9.

111. Nagabhushan RM, Shetty AP, Dumpa SR, Subramanian B, Kanna RM, Shanmuganathan R. Effectiveness and Safety of Batroxobin, Tranexamic Acid and a Combination in Reduction of Blood Loss in Lumbar Spinal Fusion Surgery. SPINE. 2018;43(5):E267-E73.

112. Nct. Tranexamic Acid (TXA) Versus Epsilon Aminocaproic Acid (EACA) Versus Placebo for Spine Surgery. <https://clinicaltrialsgov/show/NCT00958581>. 2009.

113. Nct. Does Tranexamic Acid Decrease Blood Loss in Pediatric Idiopathic Scoliosis Surgery? <https://clinicaltrialsgov/show/NCT01813058>. 2013.

114. Nct. CLOSED SUCTION DRAIN VS. NO DRAIN IN ADOLESCENTS UNDERGOING PEDICLE SCREW INSTRUMENTATION FOR IDIOPATHIC SCOLIOSIS (DAISY 2018). A Randomized Clinical Trial. <https://clinicaltrialsgov/show/NCT03729947>. 2018.

115. Nct. Posterior Column Spinal Osteotomies in the Treatment of Adolescent Idiopathic Scoliosis. <https://clinicaltrialsgov/show/NCT05379868>. 2022.

116. Newall M, Hamdan TA, Lui DF, Ajayi B, Bishop T, Weil S. Tranexamic acid use in a patient with sickle cell disease undergoing posterior scoliosis correction surgery: safely mitigating bleeding and vaso-occlusive crises. J Surg Case Rep. 2021;2021(1):rjaa559.

117. Ng BK, Chau WW, Hung AL, Hui AC, Lam TP, Cheng JC. Use of Tranexamic Acid (TXA) on reducing blood loss during scoliosis surgery in Chinese adolescents. Scoliosis. 2015;10:28.

118. Niemeyer MJS, Jochems D, Houwert RM, van Es MA, Leenen LPH, van Wessem KJP. Mortality in polytrauma patients with moderate to severe TBI on par with isolated TBI patients: TBI as last frontier in polytrauma patients. Injury. 2022;53(4):1443-8.

119. Nugent M, Tarrant RC, Queally JM, Sheeran P, Moore DP, Kiely PJ. Influence of curve magnitude and other variables on operative time, blood loss and transfusion requirements in adolescent idiopathic scoliosis. IRISH JOURNAL OF MEDICAL SCIENCE. 2016;185(2):513-20.

120. O'Donnell C, Michael N, Bloch N, Erickson M, Garg S. Strategies to Minimize Blood Loss and Transfusion in Pediatric Spine Surgery. JBJS reviews. 2017;5(5):e1-e.

121. O'Donoghue K, Wake S, Nathanson M. Patient blood management during major spinal deformity corrective surgery. Anaesthesia. 2020;75:47.

122. Oetgen ME, Litrenta J. Perioperative Blood Management in Pediatric Spine Surgery. JOURNAL OF THE AMERICAN ACADEMY OF ORTHOPAEDIC SURGEONS. 2017;25(7):480-8.

123. Ohashi N, Ohashi M, Endo N, Kohno T. Administration of tranexamic acid to patients undergoing surgery for adolescent idiopathic scoliosis evokes pain and increases the infusion rate of remifentanil during the surgery. PLoS One. 2017;12(3):e0173622.

124. Ohrt-Nissen S, Bukhari N, Dragsted C, Gehrchen M, Johansson P, Dirks J, et al. Blood transfusion in surgical treatment of adolescent idiopathic scoliosis: One-center experience of patient blood management in 210 cases. European Spine Journal. 2017;26(2):S397-S8.

125. Ohrt-Nissen S, Bukhari N, Dragsted C, Gehrchen M, Johansson PI, Dirks J, et al. Blood transfusion in the surgical treatment of adolescent idiopathic scoliosis-a single-center experience of patient blood management in 210 cases. Transfusion. 2017;57(7):1808-17.

126. Oliveira ES, Colombarolli SG, Nascimento CS, Batista ICA, Ferreira JGG, Alvarenga DLR, et al. Increased levels of Txa2 induced by dengue virus infection in IgM positive individuals is related to the mild symptoms of dengue. Viruses. 2018;10(3).

127. Owen AR, Tibbo ME, van Wijnen AJ, Pagnano MW, Berry DJ, Abdel MP. Acquired Idiopathic Stiffness After Contemporary Total Knee Arthroplasty: Incidence, Risk Factors, and Results Over 25 Years. JOURNAL OF ARTHROPLASTY. 2021;36(8):2980-5.

128. Pactr. A Novel Adverse Effect of Tranexamic Acid in Adolescent Idiopathic Scoliosis Surgery. <https://trialsearchwhoint/Trial2aspx?TrialID=PACTR201805003406118>. 2018.

129. Palmer GM, Pirakalathanan P, Skinner AV. A multi-centre multi-national survey of anaesthetists regarding the range of anaesthetic and surgical practices for paediatric scoliosis surgery. ANAESTHESIA AND INTENSIVE CARE. 2010;38(6):1077-84.

130. Pennington Z, Cottrill E, Lubelski D, Ehresman J, Lehner K, Groves ML, et al. Clinical utility of enhanced recovery after surgery pathways in pediatric spinal deformity surgery: systematic review of the literature. JOURNAL OF NEUROSURGERY-PEDIATRICS. 2021;27(2):225-38.

131. Pennington Z, Ehresman J, Westbroek EM, Lubelski D, Cottrill E, Sciubba DM. Interventions to minimize blood loss and transfusion risk in spine surgery: A narrative review. CLINICAL NEUROLOGY AND NEUROSURGERY. 2020;196.

132. Peters A, Verma K, Diefenbach C, Hoelscher CM, Huncke TK, Boenigk K, et al. Preoperative autologous blood donation does not affect pre-incision hematocrit in ais patients. A retrospective cohort of a prospective randomized trial. Spine journal. 2013;13(9):110S‐1S.

133. Pico J, Sola C, Saour AC, Chapron K, Coruble L, Bringuier S, et al. Enhanced recovery after surgery (ERAS) for adolescent idiopathic scoliosis: Standardisation of care improves patient outcomes. Anaesthesia Critical Care and Pain Medicine. 2022;41(5).

134. Prasad N, Jain A, Bronheim RS, Marrache M, Njoku DB, Sponseller PD. Elevated preoperative blood pressure and its relationship to intraoperative mean arterial pressure and blood loss in posterior spinal fusion for adolescent idiopathic scoliosis. European Journal of Orthopaedic Surgery and Traumatology. 2024;34(1):339-45.

135. Ramgren B, Drake M, Andsberg G. Aneurysm rupture during thrombectomy whattodo. Neuroradiology. 2017;59(1):S97-S8.

136. Rajanigandha V, Saranya S, Anoop P. Factors Affecting Intraoperative Blood Loss in Scoliosis Surgery: An Observational Cross-sectional Study. Journal of Clinical and Diagnostic Research. 2023;17(4):UC27-UC30.

137. Saleh AN, Mostafa RH. Increased nociception following administration of different doses of tranexamic acid in adolescent idiopathic scoliosis surgery. Open anesthesiology journal. 2018;12(1):61‐8.

138. Sarwahi V, Horn JJ, Kulkarni PM, Wollowick AL, Lo Y, Gambassi M, et al. Minimally invasive surgery in patients with adolescent idiopathic scoliosis. Clinical Spine Surgery. 2016;29(8):331-40.

139. Sarwahi V, Visahan K, Hasan S, Patil A, Grunfeld M, Atlas A, et al. Single Long-Incision Minimally Invasive Surgery. Spine. 2024;49(5):356-63.

140. Shackelford SA, del Junco DJ, Riesberg JC, Powell D, Mazuchowski EL, Kotwal RS, et al. Case-control analysis of prehospital death and prolonged field care survival during recent US military combat operations. Journal of Trauma and Acute Care Surgery. 2021;91(2):S186-S93.

141. Shchederkina IO, Livshitz MI, Chmutin GE, Zokhidov ZU, Oleynikov BI, Musa G. Reperfusion Therapy in Pediatric Ischemic Stroke: the Pediatric Thrombolysis Protocol at the Primary Center of Pediatric Stroke in Moscow. Latin American Journal of Pharmacy. 2022;41(Special Issue):195-202.

142. Shrestha IK, Ruan T-Y, Lin L, Tan M, Na X-Q, Qu Q-C, et al. The efficacy and safety of high-dose tranexamic acid in adolescent idiopathic scoliosis: a meta-analysis. JOURNAL OF ORTHOPAEDIC SURGERY AND RESEARCH. 2021;16(1).

143. Soini V, Syvänen J, Helenius L, Raitio A, Helenius I. Health-related quality of life after segmental pedicle screw instrumentation: a matched comparison of patients with neuromuscular and adolescent idiopathic scoliosis. Acta Orthopaedica. 2023;94:165-70.

144. Soliman HAG, Beausejour M, Joncas J, Roy-Beaudry M, Barchi S, Mac-Thiong JM, et al. Predicting lowest hemoglobin level and risk of blood transfusion in spinal fusion surgery for adolescent idiopathic scoliosis. European Spine Journal. 2019;28(6):1342-8.

145. Song J, Sun J, Sun Y, Liu Y, Gu X, Ma Z. Risk factors of massive introperative blood loss in posterior spinal fusion for adolescent idiopathic scoliosis. National Medical Journal of China. 2021;101(14):1002-8.

146. Stein AL, Roessler J, Braun J, Sprengel K, Beeler PE, Spahn DR, et al. Impact of a goal-directed factor-based coagulation management on thromboembolic events following major trauma. SCANDINAVIAN JOURNAL OF TRAUMA RESUSCITATION & EMERGENCY MEDICINE. 2019;27(1).

147. Sui W-y, Ye F, Yang J-l. Efficacy of tranexamic acid in reducing allogeneic blood products in adolescent idiopathic scoliosis surgery. BMC MUSCULOSKELETAL DISORDERS. 2016;17.

148. Süzer MA. PERIOPERATIVE MANAGEMENT IN SCOLIOSIS SURGERY: SPINAL MUSCULAR ATROPHY TYPE II/III VERSUS ADOLESCENT IDIOPATHIC SCOLIOSIS. Journal of Turkish Spinal Surgery. 2023;34(3):118-23.

149. Tan GM, Austin T, Garg S, Cohen MN. A multimodal approach reduced allogeneic blood transfusions by over 50% in pediatric posterior spinal fusion (PSF) surgeries. Anesthesia and Analgesia. 2018;127(3):2-3.

150. Tan H, Pan S, Wei C, Chen Z, Chen T. Comparative efficacy and safety of different hemostatic medications during spinal surgery: A network meta-analysis. MEDICINE. 2023;102(9).

151. Thompson GH, Florentino-Pineda I, Poe-Kochert C. The role of Amicar in decreasing perioperative blood loss in idiopathic scoliosis. SPINE. 2005;30(17):S94-S9.

152. Toombs C, Verma K, Lonner BS, Feldman D, Errico T. Preliminary Analysis of Factors Associated with Blood Loss in Neuromuscular Scoliosis Surgery. Bulletin of the Hospital for Joint Disease (2013). 2018;76(3):207-15.

153. Tumber S, Bacon A, Stondell C, Tafoya S, Taylor SL, Javidan Y, et al. High- versus low-dose tranexamic acid as part of a Patient Blood Management strategy for reducing blood loss in patients undergoing surgery for adolescent idiopathic scoliosis. Spine Deform. 2022;10(1):107-13.

154. Uehara M, Takahashi J, Ikegami S, Kuraishi S, Futatsugi T, Oba H, et al. Correlation of Lower Instrumented Vertebra with Spinal Mobility and Health-related Quality of Life after Posterior Spinal Fusion for Adolescent Idiopathic Scoliosis. Clinical Spine Surgery. 2019;32(7):E326-E9.

155. Urbanski W, Markowski P, Zaluski R, Kokaveshi A, Morasiewicz P. Direct Vertebral Rotation (DVR) Does Not Improve Clinical and Radiological Results Compared to Differential Rod Contouring (DRC) in Patients Treated Surgically for Idiopathic Scoliosis. Journal of Clinical Medicine. 2023;12(12).

156. Van Haren RM, Valle EJ, Thorson CM, Jouria JM, Busko AM, Guarch GA, et al. Hypercoagulability and other risk factors in trauma intensive care unit patients with venous thromboembolism. J Trauma Acute Care Surg. 2014;76(2):443-9.

157. van Popta D, Stephenson J, Patel D, Verma R. The pattern of blood loss in adolescent idiopathic scoliosis. SPINE JOURNAL. 2014;14(12):2938-45.

158. van Wessem KJP, Jochems D, Leenen LPH. The effect of prehospital tranexamic acid on outcome in polytrauma patients with associated severe brain injury. Eur J Trauma Emerg Surg. 2022;48(3):1589-99.

159. Vasan PK, Rajasekaran S, Viswanathan VK, Shetty AP, Kanna RM. Is fresh, leucodepleted, whole blood transfusion superior to blood component transfusion in pediatric patients undergoing spinal deformity surgeries? A prospective, randomized study analyzing postoperative serological parameters and clinical recovery. European Spine Journal. 2021;30(7):1943-9.

160. Verma K, Errico T, Diefenbach C, Hoelscher C, Peters A, Dryer J, et al. The relative efficacy of antifibrinolytics in adolescent idiopathic scoliosis: a prospective randomized trial. Journal of bone and joint surgery American volume. 2014;96(10):e80.

161. Verma K, Errico TJ, Vaz KM, Lonner BS. A prospective, randomized, double-blinded single-site control study comparing blood loss prevention of tranexamic acid (TXA) to epsilon aminocaproic acid (EACA) for corrective spinal surgery. BMC surgery. 2010;10:13.

162. Von kier S, Davies A, Larkin C, Maher K, Rogers R. Dedicated interventional blood management service reduces need for blood transfusion in thoraco-lumbar posterior instrumented fusion for adolescent idiopathic scoliosis. Anesthesia and Analgesia. 2021;133(3 SUPPL 1):37.

163. Wada T, Shiraishi A, Gando S, Yamakawa K, Fujishima S, Saitoh D, et al. Pathophysiology of Coagulopathy Induced by Traumatic Brain Injury Is Identical to That of Disseminated Intravascular Coagulation With Hyperfibrinolysis. Frontiers in Medicine. 2021;8.

164. Wagala NN, Marasigan JAM, Mian HM, Schwend RM. Operative time in adolescent idiopathic scoliosis surgery: a need for a standard definition. JOURNAL OF PEDIATRIC ORTHOPAEDICS-PART B. 2021;30(3):205-10.

165. Wahlquist S, Wongworawat M, Nelson S. When Does Intraoperative Blood Loss Occur During Pediatric Scoliosis Correction? Spine Deformity. 2017;5(6):387-91.

166. Wang L, Liu J, Song X, Luo M, Chen Y. Hidden blood loss in adolescent idiopathic scoliosis patients undergoing posterior spinal fusion surgery: a retrospective study of 765 cases at a single centre. BMC Musculoskelet Disord. 2021;22(1):794.

167. Wang N, Zhou Q, Cehn H, Pu X. The Efficacy and Safety of Tranexamic Acid in Adolescent Idiopathic Scoliosis Correction Surgery. Chinese Journal of Spine and Spinal Cord. 2024;34(01):14-9.

168. Wirtz MR, Muller MCA, Johansson PI, Brohi K, Stanworth SJ, Meagele M, et al. Incidence, risk factors and outcome of late hypercoagulability in trauma as measured by viscoelastic haemostatic assays. Intensive Care Medicine Experimental. 2018;6.

169. Wolff S, Habboubi K, Sebaaly A, Moreau PE, Miladi L, Riouallon G. Correction of adult spinal deformity with a minimally invasive fusionless bipolar construct: Preliminary results. ORTHOPAEDICS & TRAUMATOLOGY-SURGERY & RESEARCH. 2019;105(6):1149-55.

170. Wu M, Dai Z, Liang Y, Liu X, Zheng X, Zhang W, et al. Respiratory variation in the internal jugular vein does not predict fluid responsiveness in the prone position during adolescent idiopathic scoliosis surgery: a prospective cohort study. BMC ANESTHESIOLOGY. 2023;23(1).

171. Xie JM, Li T, Wang Y, Zhang Y, Bi N, Zhao Z, et al. Different dose regimens of tranexamic acid reduces perioperative blood loss and blood transfusion in adolescent idiopathic scoliosis patients: a prospective, randomized control study. Spine journal. 2015;15(10 SUPPL. 1):S176.

172. Xu C, Wu A, Yue Y. Which is more effective in adolescent idiopathic scoliosis surgery: batroxobin, tranexamic acid or a combination? Archives of orthopaedic and trauma surgery. 2012;132(1):25‐31.

173. Yagi M, Hasegawa J, Nagoshi N, Iizuka S, Kaneko S, Fukuda K, et al. Does the intraoperative tranexamic acid decrease operative blood loss during posterior spinal fusion for treatment of adolescent idiopathic scoliosis? Spine (Phila Pa 1976). 2012;37(21):E1336-42.

174. Yan H, Liu M, Zhang H, Yan J. The Effect of Different Intravenous Administration Methods of Tranexamic Acid on Intraoperative Blood Loss in Posterior Correction Surgery for Adolescent Idiopathic Scoliosis. Journal of Spinal Surgery. 2021;19(06):367-71.

175. Yang YJ, Huang X, Gao XN, Xia B, Gao JB, Wang C, et al. An Optimized Enhanced Recovery After Surgery (ERAS) Pathway Improved Patient Care in Adolescent Idiopathic Scoliosis Surgery: A Retrospective Cohort Study. World Neurosurgery. 2021;145:e224-e32.

176. Ye Z, Zong Z, Zhong X, Jia Y, Jiang R, Yang H, et al. Characterization of Combined Blast- and Fragment-Induced Pelvic Injuries and Hemostatic Resuscitation in Rabbits. J Surg Res. 2023;285:158-67.

177. Yunus SNZ. Tranexamic Acid in Peadiatric Scoliosis Surgery (Tripss): A Prospective Randomised Trial Comparing High Dose and Low Dose Tranexamic Acid in Adolescent Idiopathic Scoliosis Undergoing Posterior Spinal Fusion2019 2019.

178. Zhang N, Pan Z, Zhang N, Wang H. Effect of high-dose and standard-dose tranexamic acid on coagulation function in patients with spinal orthopedics. Medical Journal of National Defending Forces in Southwest China. 2020;30(05):431-4.

179. Zhang Z, Wang L-N, Yang X, Liu L-M, Xiu P, Zhou Z-J, et al. The effect of multiple-dose oral versus intravenous tranexamic acid in reducing postoperative blood loss and transfusion rate after adolescent scoliosis surgery: a randomized controlled trial. SPINE JOURNAL. 2021;21(2):312-20.

180. Zhang Z, Yang X, Wang L, Song Y. Retrospective Study on the Effect of Multiple Oral and Intravenous Administration of Tranexamic Acid on Reducing Postoperative Blood Loss and Transfusion Rate in Adolescent Idiopathic Scoliosis Correction Surgery. Orthopedics. 2023;14(01):46-51.

181. Zhong J, Cao K, Wang B, Zhou X, Lin N, Lu H. The Perioperative Efficacy and Safety of Tranexamic Acid in Adolescent Idiopathic Scoliosis. World Neurosurg. 2019;129:e726-e32.

182. Zielinski MD, Schrager JJ, Johnson P, Stubbs JR, Polites S, Zietlow SP, et al. Multicenter Comparison of Emergency Release Group A versus AB Plasma in Blunt-Injured Trauma Patients. Clinical and Translational Science. 2015;8(1):43-7.
