# Supplementary material for: Comparative efficacy and safety of high-dose versus low-dose tranexamic acid in adolescent idiopathic scoliosis: A systematic review and meta-analysis
Source: PLoS One. 2025 Apr 1;20(4):e0320391. doi: 10.1371/journal.pone.0320391 (PMC11960895; doi:10.1371/journal.pone.0320391)
Supplement: S4 Table — (DOCX) [file pone.0320391.s004.docx]

**Data Extraction Table for Systematic Review and Meta-Analysis (Extracted by Xin Liu and Zhong Ma on April 15, 2024)**

| **author（year）** | **Dose of TXA**  **(loading + maintenance)**  **H VS L** | **Intraoperative blood loss**  **H VS L** | **Operation time**  **H VS L** | **Transfusion rate**  **H VS L** | **Thromboembolic events**  **H VS L** |
| --- | --- | --- | --- | --- | --- |
| Tumber (2022) | 30 mg/kg+10 mg/kg/h;  <30 mg/kg+10 mg/kg/h | 1035.2±674.9;  1881.6±1505.1 | 299.2±87.5;  350.3±77.5 | 25;66 | None; None |
| Hasan (2021) | 30 mg/kg+10 mg/kg/h;  10 mg/kg+1 mg/kg/h | 844.9±384.9;  815.1±383.9 | 130.0±37.7;  123.5±37.7 | 1;1 | None; None |
| Zhang (2020) | 100 mg/kg+10 mg/kg/h;  10 mg/kg+10 mg/kg/h | 392.00±35.49;  652.00±72.72 | N/A | 5;6 | None; None |
| Saleh (2018) | 50 mg/kg+20 mg/kg/h;  10 mg/kg+10 mg/kg/h | 181.25±18.7;  229±22.44 | 128.3±7.6;  188±13.41 | N/A | None; None |
| Johnson (2017) | 50 mg/kg+5 mg/kg/h;  10 mg/kg+1 mg/kg/h | 695±372;  968±756 | 216±42;  216±54 | 9;25 | None; None |
| Grant (2009) | 20 mg/kg+10 mg/kg/h;  10 mg/kg+1 mg/kg/h | N/A | 432±66;  384±96 | 5;13 | None; None |
